# Development of photodynamic therapy regimens that control primary tumor growth and inhibit secondary disease

**DOI:** 10.1007/s00262-014-1633-9

**Published:** 2014-11-11

**Authors:** Madeeha Shams, Barbara Owczarczak, Patricia Manderscheid-Kern, David A. Bellnier, Sandra O. Gollnick

**Affiliations:** Department of Cell Stress Biology, PDT Center, Roswell Park Cancer Institute, Elm & Carlton Sts, Buffalo, NY 14263 USA

**Keywords:** Photodynamic therapy, Anti-tumor immunity, Combination therapy, Cancer

## Abstract

Effective therapy for advanced cancer often requires treatment of both primary tumors and systemic disease that may not be apparent at initial diagnosis. Numerous studies have shown that stimulation of the host immune system can result in the generation of anti-tumor immune responses capable of controlling metastatic tumor growth. Thus, there is interest in the development of combination therapies that both control primary tumor growth and stimulate anti-tumor immunity for control of metastatic disease and subsequent tumor growth. Photodynamic therapy (PDT) is an FDA-approved anticancer modality that has been shown to enhance anti-tumor immunity. Augmentation of anti-tumor immunity by PDT is regimen dependent, and PDT regimens that enhance anti-tumor immunity have been defined. Unfortunately, these regimens have limited ability to control primary tumor growth. Therefore, a two-step combination therapy was devised in which a tumor-controlling PDT regimen was combined with an immune-enhancing PDT regimen. To determine whether the two-step combination therapy enhanced anti-tumor immunity, resistance to subsequent tumor challenge and T cell activation and function was measured. The ability to control distant disease was also determined. The results showed that the novel combination therapy stimulated anti-tumor immunity while retaining the ability to inhibit primary tumor growth of both murine colon (Colon26-HA) and mammary (4T1) carcinomas. The combination therapy resulted in enhanced tumor-specific T cell activation and controlled metastatic tumor growth. These results suggest that PDT may be an effective adjuvant for therapies that fail to stimulate the host anti-tumor immune response.

## Introduction

Cancer is a multi-faceted disease, and although therapeutic outcomes have improved over the decades and single treatment modalities are generally effective at controlling the primary tumor, they have little effect on distant disease. This has led to the development of combination therapies that strive to both control primary tumor growth and activate the host immune system to combat distant disease. Photodynamic therapy (PDT) is an FDA-approved anticancer modality used for treatment of early stage disease and palliation of advanced disease [1]. Treatment is carried out by systemic administration of a photosensitizer that is activated locally with tissue-penetrating visible light, resulting in tumor destruction via oxygen-dependent direct tumor cell cytotoxicity, vascular damage, and the initiation of acute local and systemic inflammation. The photosensitizer dose, light dose, and light dose-rate define the PDT regimen [2]. Numerous preclinical and clinical studies have shown that PDT efficacy is dependent upon the presence of an intact immune system [1] and that certain PDT regimens can enhance anti-tumor immunity [3, 4].

PDT-enhanced anti-tumor immunity is mediated by CD8^+^ cytotoxic T cells (CTLs) [3, 4] and is accompanied by enhanced CTL-mediated tumor cytotoxicity, formation of immune memory cells, and resistance to subsequent tumor growth [5]. CD8^+^ T cell depletion or tumor cell loss of molecules needed for CD8^+^ T cell recognition [major histocompatibility complex (MHC) class I molecules] result in reduced PDT efficacy [5–7]. PDT-activated CD8^+^ T cells also play a critical role in PDT-mediated control of secondary disease [8]. PDT enhancement of CTL activation and anti-tumor immunity is dependent upon induction of acute inflammation [5].

PDT-induced acute inflammation is characterized by rapid migration of neutrophils into the treated tumor bed and tumor-draining lymph nodes and enhanced expression of pro-inflammatory chemokines and cytokines [9, 10]. The degree of acute inflammation induced by PDT is regimen dependent and correlates with the degree of vascular damage induced [2]. Henderson et al. [2] demonstrated that PDT regimens that result in rapid cell death (within 1 h of treatment) and maximal tissue damage cause minimal acute inflammation, presumably because of vascular shut down, which prevents neutrophil infiltration and systemic release of cytokines. In contrast, regimens that cause diffuse tumor damage permit neutrophil infiltration and allow for expression and release of inflammatory mediators critical for enhancement of anti-tumor immunity. However, the diffuse tumor damage caused by these regimens resulted in less effective long-term tumor control.

Combination therapies take advantage of the complementary action of the individual components, thereby potentiating the therapeutic effect. We hypothesized that it would be possible to devise a PDT-only therapy using distinctly different, but complimentary, treatment regimens that in combination would enhanced anti-tumor immunity as well as provide effective control of primary tumor growth. To test this hypothesis, we devised a multi-step local treatment program that was comprised of an immune-enhancing PDT regimen followed by a tumor ablation PDT regimen. Results presented here demonstrate that this program was able to control the growth of murine colon and mammary tumors and to promote anti-tumor immunity that was effective against distant disease. These studies demonstrate that PDT-only therapies can be developed that are effective as both a local therapy for control of primary disease and a systemic therapy for treatment of distant disease.

## Materials and methods

### Animals and tumor system

Pathogen-free BALB/c mice were obtained from the NCI; *scid* mice were obtained from the Roswell Park Department of Laboratory Animal Resources. All mice were female, of BALB/c background, and were housed in microisolator cages in a laminar flow unit under ambient light. Six- to ten-week-old animals were inoculated subcutaneously on either the right shoulder with 10^6^ Colo26 (murine colon carcinoma) or Colo26 cells transfected with hemagglutinin (HA) cDNA (Colo26-HA; [11]) or in the mammary fat pad with 5 × 10^5^ 4T1 mammary carcinoma cells. Intravenous challenge with tumor cells was performed by injection of exponentially growing tumor cells. The Roswell Park Cancer Institutional Animal Care and Use Committee (IACUC) approved all procedures carried out in this study.

### Reagents

Clinical-grade, pyrogen-free 2-[1-hexyloxyethyl]-2-devinyl pyropheophorbide-a (HPPH) was obtained from the Roswell Park Pharmacy (Buffalo, NY) and reconstituted to 0.4 μM in pyrogen-free 5 % dextrose in water (D5W; Baxter Corp., Deerfield, IL). Porfimer sodium (PII) was obtained from Pinnacle Biologics (Bannockburn, IL) and reconstituted in D5W.

### *In vivo* PDT treatment

Tumor-bearing mice were injected in the tail vein with 0.4 μmol/kg HPPH or 5 mg/kg PII, followed 18–24 h later by illumination to a total light dose of 48 J/cm^2^ or 132 J/cm^2^ delivered at a light dose-rate of 14 mW/cm^2^ as previously described [2, 5]. Control mice were treated with photosensitizer or light alone. Mice receiving a combination PDT regimen were treated initially with 0.4 μmol/kg HPPH or 5 mg/kg PII followed 18–24 h later by light dose of 48 J/cm^2^ given at 14 mW/cm^2^; 9 days later, mice were again injected with photosensitizer and tumors were illuminated with light at a dose of 132 J/cm^2^ given at 14 mW/cm^2^.

### Transmission of PDT-activating light through tumor tissue

Noninvasive reflectance spectroscopy was used to measure the penetration of 665-nm light through subcutaneously implanted Colo26-HA tumors. Light attenuation was determined as previously described [12–15]. Briefly, the diffuse fluence *Φ* at 665 nm was measured at increasing probe separations. The total attenuation *α* is the slope of ln(rΦ) plotted against *r*, where *r* is the probe separation in mm and *Φ* is the diffuse 665 nm fluence escaping from the tumor at *r* mm from the light source fiber.

### Tumor response determination

Following treatment, orthogonal diameters of tumors were measured once every 2 days with calipers as previously described [2]. Animals were considered cured if they remained tumor free for 60 days after PDT. Treatment with light alone, at either dose, had no effect on the growth of Colo26-HA or 4T1 tumors.

### Flow cytometry

Auxiliary tumor-draining lymph nodes (TDLNs) or tumors were harvested at the indicated time points, and single cell suspensions were generated [16]. Cells were stained with a panel of monoclonal antibodies (MAbs) to detect specific cell surface antigens (CD8, CD11b, CD25, CD44, CD45, CD69, Ly6C, and Ly6G), as previously described [16]. At least five mice per group were analyzed. For the determination of the absolute number of specific cell populations, the percentage of each population was multiplied by the number of cells recovered from the respective TDLN or tumor.

### *In Vivo* cytotoxicity assay

*In vivo* cytotoxic assays were performed as previously described [17]. Briefly, TDLNs were harvested from treated mice 2 days post-PDT and adoptively transferred into naive BALB/c hosts as previously described [5]. Red blood cell depleted spleen cells isolated from naïve mice were used as target cells for the assay. Target cells were prepared by labeling a single cell suspension with PKH26 dye (Sigma, St. Louis, MO) for 4 min at 2 μM according to the manufacturer’s instructions. The resulting red population was separated in two equal fractions one of which was labeled with 0.1 µM carboxyfluorescein triacetate, succinimidyl ester (CFDA-SE, referred to as CFSE, Molecular Probes) and the other with 1 µM CFSE for 2 min. The lower-intensity-labeled fraction was pulsed for 1.5 h with an irrelevant peptide PA1 [18]; the high-intensity-labeled fraction was pulsed with the Colo26-specific peptide AH1 [19]. Equal cell numbers from both populations were admixed and injected into recipient mice. Twenty-four hours later peripheral blood was collected, RBC were lysed, cells were fixed, and then run on a FACS Calibur flow cytometer. Target cells were distinguished from host cells based on PKH26 staining; PA1 peptide- and AH1 peptide-loaded cells were distinguished from one another based on CFSE staining. Gating on PKH26^+^ cells, specific cytotoxicity was calculated according to the formula:

$$ \left( {1 - \left[ {{{\% {\text{ AH}}1{\text{ cells}}} \mathord{\left/ {\vphantom {{\% {\text{ AH}}1{\text{ cells}}} {\% {\text{ positive PA1 cells}}}}} \right. \kern-0pt} {\% {\text{ positive PA1 cells}}}}} \right]} \right) \times 100\,\% . $$

### Assessment of lung tumor growth

4T1 tumor-bearing mice were treated with PII; 18 h following PII injection, tumors were treated with immune-enhancing PDT. After 9 days, tumors that had been treated with immune-enhancing PDT regimens were either surgically removed or treated with tumor-controlling PDT. Tumors at an equivalent size to those treated 9 days following the immune-enhancing PDT regimen were treated with PII alone, surgically removed or treated with the tumor-controlling PDT regimen. Fourteen days after the final treatment, the presence of tumors was determined by injection of 1 ml of 15 % India ink (diluted in PBS) which was injected via an incision in the trachea. The lungs were removed from the rib cage, weighed, and placed in Fekete’s fixative (61 % ethanol, 3.2 % formaldehyde, 4.1 % acetic acid); lung tumors were counted under a dissecting microscope [20].

### Statistical evaluation

All measured values are presented as mean ± SEM. Student’s *t* test with Welch’s correction was used for comparison between groups in all of the experiments, with the exception of tumor response experiments, which were analyzed using Log-rank (Mantel-Cox) analysis of survival curves. The term “significant” in the text represents a *P* value equal to or less than 0.05.

## Results

### Long-term tumor growth control following combination PDT

Our previous studies indicated that treatment regimens that led to optimal long-term tumor response elicited only marginal inflammation and limited enhancement of anti-tumor immunity [2, 5]. These treatment regimens also ablated the tumor vasculature, and treated tumors showed widespread areas of apoptosis that was accompanied by prolonged caspase 3 activation. In contrast, regimens that elicited acute inflammation and strong anti-tumor immunity preserved the tumor vasculature, resulted in minimal long-term tumor growth control, limited areas of apoptosis, and transient caspase 3 activation. Therefore, we refer to these treatments as immune enhancing, while those regimens that effectively control tumor growth, but result in minimal enhancement of anti-tumor immunity, are referred to as tumor-controlling regimens. We hypothesized that treating tumors with an immune-enhancing PDT regimen followed by a tumor-controlling PDT regimen could lead to enhancement of anti-tumor immunity, while retaining effective control of primary tumor growth. To test this hypothesis, a combination treatment regimen was devised in which Colo26-HA tumor-bearing BALB/c mice were treated with a HPPH-PDT regimen known to lead to enhanced anti-tumor immunity (0.4 μmoles/kg HPPH followed 18 h later by illumination with 665 nm light for a total dose of 48 J/cm^2^ [5]). Following illumination, mice were rested for 9 days; on the ninth day, mice were injected with HPPH. On day 10 following the first treatment, tumors were treated with a tumor control treatment regimen (illumination with 665 nm light for a total dose of 132 J/cm^2^ given [5]).

PDT efficacy depends on the intensity and distribution of light in the tumor. We were concerned that the initial PDT exposure might change the optical properties of the tumor tissue and perturb the effectiveness of the subsequent PDT exposure. Therefore, we measured light transmission through Colo26-HA tumors immediately before both the first and the second PDT treatments and compared their calculated optical penetration depths (characterized by δ, the distance over which diffuse light decreases in intensity to 1/e or 37 % of its initial value) through tumor tissue. Light penetration measured immediately before the second PDT treatment was slightly greater than that determined before the first PDT treatment (*δ*_PDT1_ = 2.80 mm, *δ*_PDT2_ = 3.06 mm), but that difference was not statistically significant. Thus, prior treatment with an immune-enhancing PDT regimen does not alter the optical properties of the tumor tissue.

**Fig. 1:**
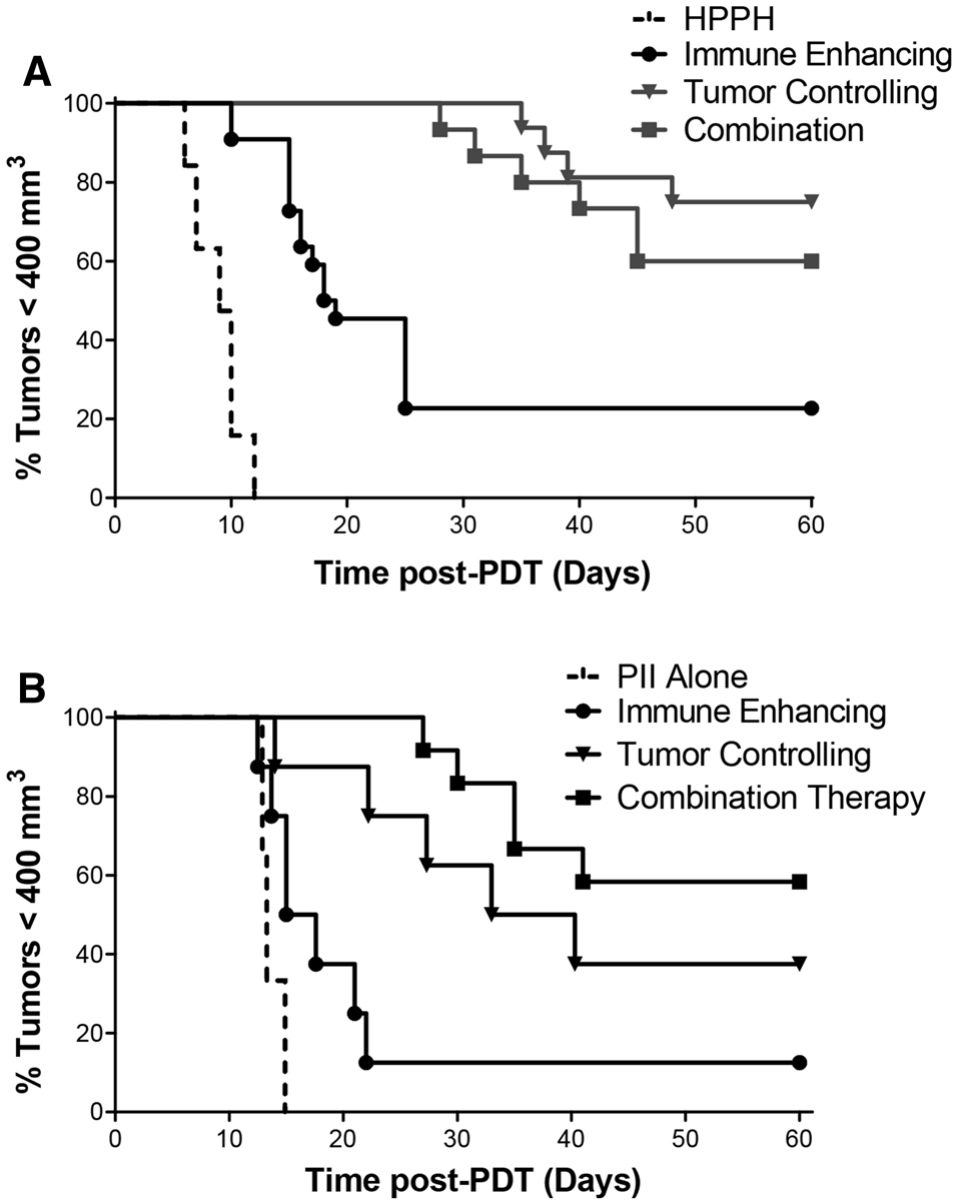
Long-term tumor growth controlfollowing combination therapy. Colo26-HA tumor-bearing mice were injected systemically with HPPH (**a**; 0.4 μmol/kg) or PII (**b**; 5 mg/kg); 18–24 h later, mice were subjected to PDT at a total dose of 48 J/cm^2^. Nine days later, treated mice were randomly divided into two groups. One group was injected systemically with HPPH (**a**; 0.4 μmol/kg) or PII (**b**; 5 mg/kg); tumor growth was monitored in the second group, which represents the immune-enhancing treatment group. Control mice bearing Colo26-HA tumors of equal size to those regrowing in mice that had been previously treated with PDT at a dose of 48 J/cm^2^ were also injected systemically with HPPH or PII; 18–24 h later, control mice were left untreated (HPPH or PII alone) or treated with PDT (132 J/cm^2^; tumor-controlling regimen). Mice that had been previously treated with PDT at a dose of 48 J/cm^2^ were retreated with PDT at a dose of 132 J/cm^2^ (combination). Each group contained a minimum of eight animals. Tumor growth was followed until tumors reached 400 mm^3^ or 60 days

We next tested whether prior treatment with an immune-enhancing PDT regimen altered effectiveness of tumor-controlling PDT regimens. Colo26-HA tumor-bearing mice were treated with the combination therapy (immune-enhancing PDT followed 10 days later with tumor-controlling PDT). Animals bearing tumors of an equivalent size to the regrowing tumors present in the animals treated initially with immune-enhancing PDT at the time of the second HPPH injection (9 days following delivery of the initial immune-enhancing PDT regimen) were also injected with HPPH and were treated 18 h later with the tumor control PDT regimen or were treated with 665 nm light for a total dose of 48 J/cm^2^ or 135 J/cm^2^. Tumor growth was monitored for 60 days (Fig. 1a). All treatments resulted in significant tumor control when compared to the control treatment with either HPPH or light alone (*P* ≤ 0.001); for simplicity sake, the tumor growth response of tumors treated with light alone is not depicted. The combination treatment controlled tumor growth as well as the single treatment at 132 J/cm^2^ (*P* ≤ 0.4) and significantly better than treatment with 48 J/cm^2^ (*P* ≤ 0.001). Thus, demonstrating that prior treatment of tumors with an immune-enhancing regimen did not affect tumor growth control by the optimal tumor growth control treatment regimen.

To determine whether the results obtained were limited to a specific photosensitizer, the two-step combination therapy was performed using sodium Porfimer (PII) as the photosensitizer. Colo26-HA tumor-bearing mice were treated as above except that PII (5 mg/kg) was used in place of HPPH as the photosensitizer. Colo26-HA tumors responded similarly to PII-PDT as they did to HPPH-PDT (Fig. 1b). Minimal but significant tumor efficacy was observed following treatment with the immune-enhancing PII-PDT regimen (48 J/cm^2^; *P* ≤ 0.05); treatment with either the tumor-controlling PII-PDT treatment regimen (132 J/cm^2^) or the two-step combination therapy resulted in increased tumor growth control that was significantly greater than that observed with either PII treatment alone or treatment with the low-dose PDT regimen (*P* ≤ 0.003 or *P* ≤ 0.04, respectively). There was no significant difference in tumor growth control between treatment with the high-dose PDT regimen and the two-step combination therapy.

**Fig. 2:**
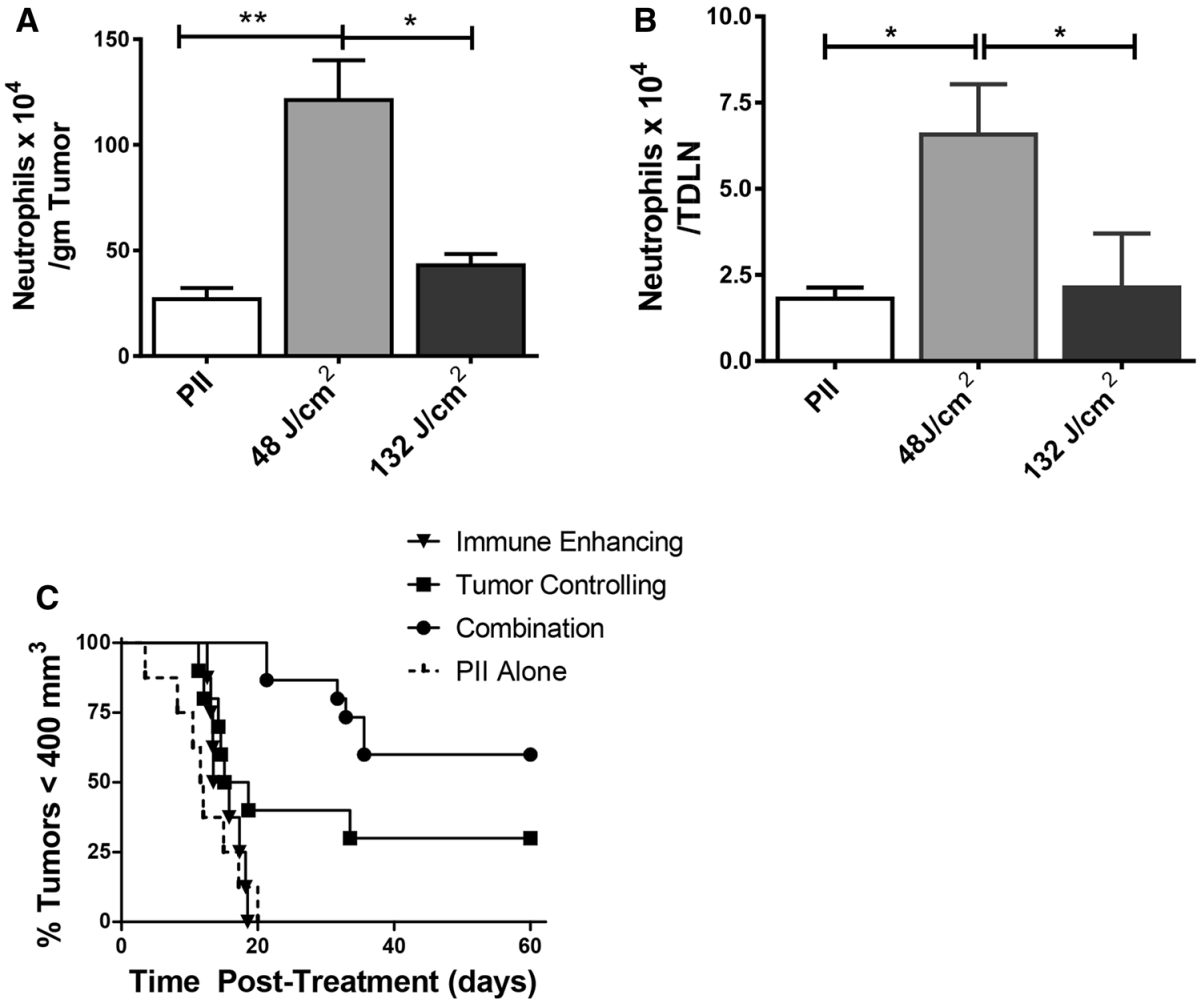
Combination therapy increases long-term tumor growth control. **a,** **b** 4T1 tumor-bearing mice were treated with PII-PDT (5 mg/kg PII) doses of 48 or 132 J/cm^2^. Control animals were treated with PII alone. Each group contained a minimum of five animals. Tumors (**a**) and tumor-draining lymph nodes (TDLN; **b**) were collected 4 h after the final treatment. Single cell suspensions were generated, and the number of neutrophils (CD45^+^CD11b^+^Ly6C^+^Ly6G^Hi^)/gm of tumor (**a**) or (CD11b^+^ Ly6C^+^Ly6G^Hi^)/TDLN (**b**) was determined. *Error bars* represent SEM. *P ≤ 0.05; **P ≤ 0.01 (**c**) 4T1 tumor-bearing mice were injected systemically with PII (5 mg/kg PII); 18–24 h later, mice were subjected to PDT at a total dose of 48 J/cm^2^. Nine days later, treated mice were randomly divided into two groups. One group was injected systemically with PII (5 mg/kg); tumor growth was monitored in the second group, which represents the immune-enhancing treatment group. Control mice bearing 4T1 tumors of equal size to those regrowing in mice that had been previously treated with PDT at a dose of 48 J/cm^2^ were also injected systemically with PII; 18–24 h later, control mice were left untreated (PII alone) or treated with PDT (132 J/cm^2^; tumor-controlling regimen). Mice that had been previously treated with PDT at a dose of 48 J/cm^2^ were retreated with PDT at a dose of 132 J/cm^2^ (combination). Each group contained a minimum of eight animals. Tumor growth was followed until tumors reached 400 mm^3^ or 60 days

The two-step combination therapy was also tested in the 4T1 mammary carcinoma model. Neutrophil mobilization into tumor tissue is a characteristic of PDT-induced acute inflammation [5, 21]; neutrophil mobilization into tumor-draining lymph nodes (TDLNs) is required for PDT enhancement of anti-tumor immunity [5]. Therefore, neutrophil mobilization into tumors and TDLNs was examined following PDT of orthotopically growing 4T1 mammary carcinomas. 4T1 tumor-bearing mice were treated with PII-PDT (5 mg/kg PII followed 18 h later by illumination with 630 nm light for a total dose of either 48 J/cm^2^ or 132 J/cm^2^ delivered at 14 mW cm^2^); the number of infiltrating neutrophils present 4 h post-treatment in the tumor bed and TDLN was determined by flow cytometry. Neutrophils were identified as those CD45^+^CD11b^+^ cells (tumor) or CD11b^+^ cells (TDLN) expressing Ly6C and high levels of Ly6G [22]. Treatment of 4T1 tumors with a PII-PDT regimen of 48 J/cm^2^ led to a significant increase in the number of neutrophils present in the treated tumor bed (Fig. 2a) and TDLN (Fig. 2b) when compared to the number of neutrophils present in tumors or TDLN of animals treated with PII alone (*P* ≤ 0.01; *P* ≤ 0.03, respectively). In contrast, treatment with 132 J/cm^2^ resulted in a negligible increase in neutrophils in either the tumor bed or TDLN (*P* ≤ 0.1). Based upon these findings, we have defined the 48 J/cm^2^ treatment as immune enhancing.

We then examined the effect of PDT regimens on 4T1 tumor growth. 4T1 tumor-bearing mice were treated as above with PII alone, light alone (630 nm for a total dose of 48 J/cm^2^ or 135 J/cm^2^ delivered at 14 mW/cm^2^) or PII-PDT (5 mg/kg PII followed 18 h later by illumination with 630 nm light for a total dose of either 48 J/cm^2^ or 132 J/cm^2^), or with a two-step combination therapy in which 4T1 tumor-bearing mice were treated with immune-enhancing PDT followed 10 days later with treatment with the higher-dose PDT regimen (5 mg/kg PII given 9 days after the first treatment, followed 18–24 h later with illumination with 630 nm light for a total dose of 132 J/cm^2^ delivered at 14 mW cm^2^). Tumor growth following treatment was monitored for 60 days or until the primary tumor reached 400 mm^3^. Treatment of 4T1 tumors with the immune-enhancing PII-PDT regimen did not lead to long-term tumor growth control (*P* ≥ 0.05 compared to treatment with PII alone); treatment with 132 J/cm^2^ resulted in marginal, but significant control of tumor growth (*P* ≤ 0.05; Fig. 2c). This was the optimal tumor growth control achieved when the fluence rate was held to 14 mW/cm^2^ (data not shown). Therefore, the 132 J/cm^2^ treatment was identified as tumor controlling. Interestingly, when the treatments were combined, there was a significant increase in long-term tumor growth control as compared to treatment with 132 J/cm^2^ (*P* ≤ 0.05).

### Combination PDT regimen leads to enhanced anti-tumor T cell activation and activity

**Fig. 3:**
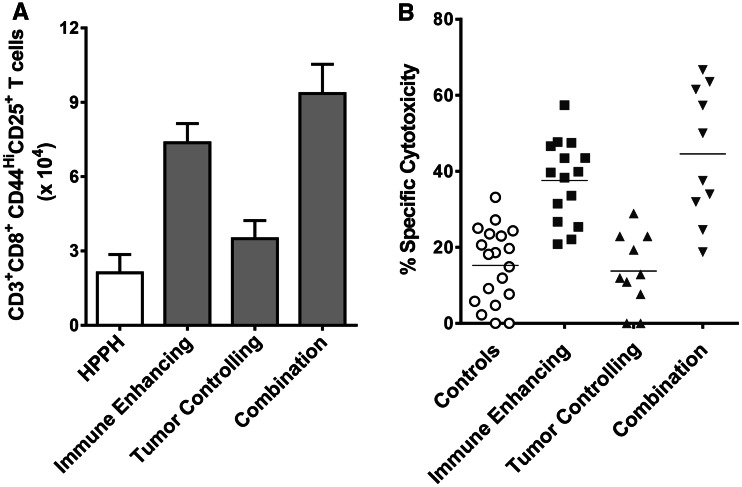
Combination therapy increases T cell activation. Colon26-HA tumor-bearing mice were treated with HPPH or HPPH-PDT (0.47 μmoles/kg HPPH) immune-enhancing (48 J/cm^2^), tumor-controlling (132 J/cm^2^) or a combination regimen of immune-enhancing treatment followed by tumor-controlling treatment as described in Fig. 1. **a** TDLNs were collected 24 h after the final treatment, and single cell suspensions were generated. The number of activated CD8^+^ T cells (CD3^+^CD8^+^CD44^Hi^CD25^+^) was determined by flow cytometry. A minimum of 14 mice were examined in each group. **b** TDLNs were collected 24 h after the final treatment, and in vivo cytotoxicity assays were performed as described in Materials and Methods. Results are presented as % specific cytotoxicity. *Each symbol* represents results obtained from an individual mouse; horizontal lines represent the median. A minimum of ten mice were examined per group

Previous studies have shown that activated T cells are susceptible to PDT [23], and it is possible that T cells activated in response to immune-enhancing PDT regimens would be deleted by subsequent PDT regimens. Therefore, we examined the effect of the combination PDT regimen on T cell activation. Colo26-HA tumor-bearing BALB/c mice were treated with immune-enhancing, tumor-controlling or combination PDT regimens. Two days following the final treatment, tumor-draining lymph node (TDLN) cells were examined by flow cytometry for the presence of activated, proliferating CD8^+^ T cells, which were defined as CD8^+^ cells that express CD25 and high levels of CD44. As observed previously [5], treatment of Colo26-HA tumors with immune-enhancing PDT regimen resulted in significantly increased numbers of activated and proliferating CD8^+^ T cells (*P* ≤ 0.01), while treatment with the tumor-controlling PDT regimen had minimal effect on the numbers of activated CD8^+^ T cells compared to CD8^+^ T cells isolated from animals treated with HPPH alone (*P* ≤ 0.2; Fig. 3a). Treatment with the combination therapy also led to significantly increased numbers of activated, proliferating CD8^+^ T cells compared to either CD8^+^ T cells isolated from animals treated with HPPH or tumor-controlling PDT (*P* ≤ 0.01); the increase in activated, proliferating CD8^+^ T cells following the combination therapy was not significantly different from the increase induced by the immune-enhancing PDT regimen (*P* ≤ 0.2). Similar results were obtained following treatment of 4T1 tumor-bearing mice (data not shown).

We have previously demonstrated that the increased number of activated and proliferating T cells following treatment of Colo26-HA tumor with immune-enhancing PDT regimens leads to increased lysis of target cells loaded with peptides derived from either the surrogate antigen, HA, or gp70, an endogenous tumor antigen expressed by Colo26-HA and the parental Colo26 tumor cell lines [5, 19]. To determine whether the increase in CD8^+^ T cell accumulation observed following the combination treatment was also associated with increase cell lysis, the ability of PDT-activated lymph node cells to eliminate target cells loaded with tumor-associated peptides was measured in vivo. TDLN cells were harvested from treated mice 2 days following the last PDT treatment and adoptively transferred into naïve BALB/c mice. Recipient mice were then challenged with an i.v. injection of cells primed with AH1 peptide, which is the immunodominant peptide from gp70 [19] or a control peptide. Recipient mice adoptively transferred with TDLN cells isolated from mice treated with immune-enhancing and combination treatment regimens exhibit significantly increased specific cytotoxicity against cells bearing tumor-specific peptides when compared to recipient mice receiving TDLN cells from control mice or mice treated with the tumor-controlling PDT regimen (Fig. 3b; *P* ≤ 0.001). As predicted and previously observed, recipient mice receiving TDLN cells from mice treated with tumor-controlling PDT regimens exhibited minimal cytotoxicity against cells expressing tumor-specific peptides. The lack of a known tumor antigen expressed by 4T1 tumors prevented us from carrying out similar assays in this model.

### Combination PDT regimen results in increased resistance to tumor challenge

**Fig. 4:**
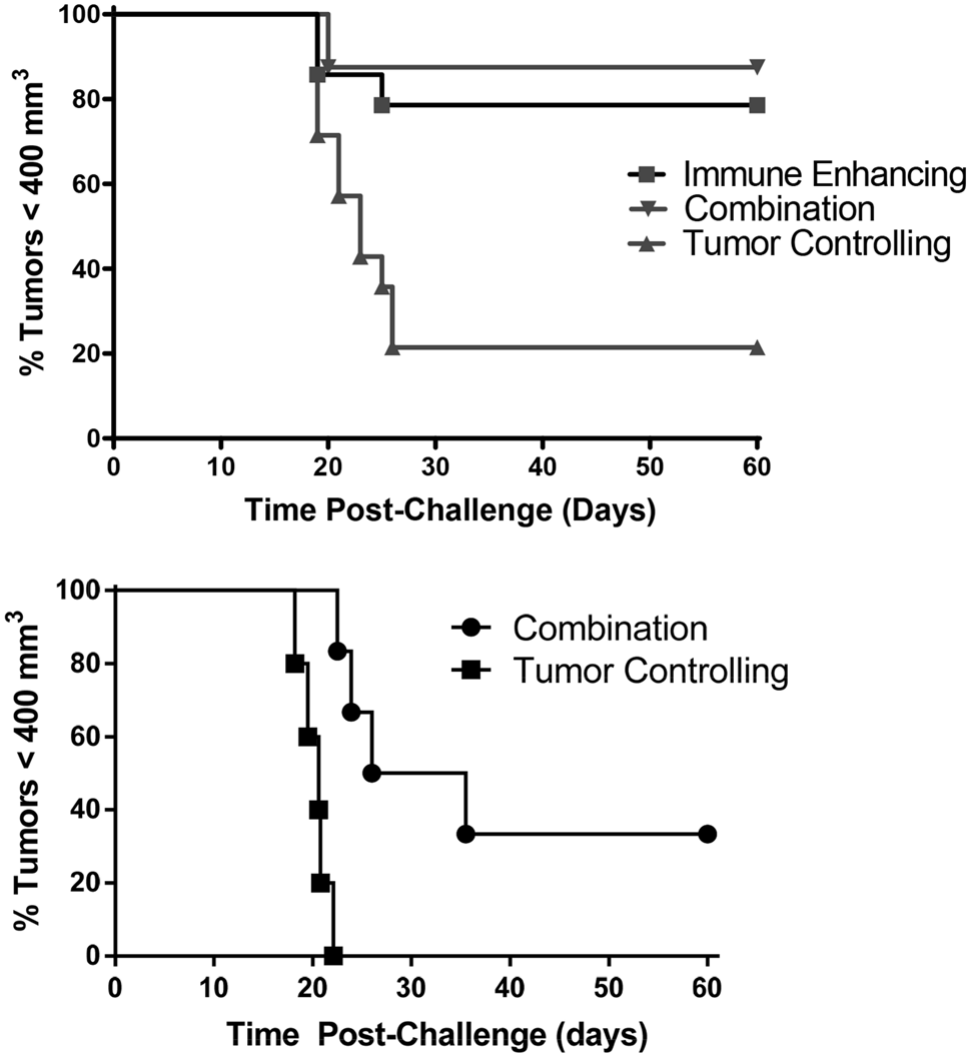
Combination therapy increases resistance to tumor challenge. Colo26-HA or 4T1 tumor-bearing mice were treated with tumor-controlling, immune-enhancing, or the combination HPPH- or PII-PDT regimens, respectively, as described in Figs. 1 and 2. Mice that remained tumor-free 40 days after treatment were challenged with live tumor cells (**a**: Colon 26 challenge; **b**: 4T1 challenge). Tumor growth was monitored for 60 days or until tumors reached 400 mm^3^

To determine whether increased cytotoxicity against tumor antigens translated to increased resistance to tumors, mice that remained tumor free for 60 days after the final PDT treatment were challenged with Colo26 cells. Colo26 cells were used in these experiments rather than Colo26-HA cells in order to validate the results obtained from the in vivo cytotoxicity experiments (Fig. 3b) and to further confirm that PDT enhancement of anti-tumor immunity was not restricted to immunity against over-expressed surrogate antigens as was demonstrated by Mroz et al. [24]. We have shown previously that the tumor response of parental Colo26 tumors and Colo26-HA tumors to HPPH-PDT is indistinguishable [5]. Colo26-HA tumor-bearing mice that remained tumor free following treatment with either immune-enhancing or combination regimens were more resistant to subsequent challenge with Colo26 cells than mice that remained tumor free following treatment with tumor--controlling PDT regimens (*P* ≤ 0.01; Fig. 4a), confirming that enhancement of anti-tumor immunity was not solely due to enhanced recognition of the surrogate antigen HA. Greater than 60 % of mice treated with the tumor-controlling PDT regimen remained tumor free for 60 days; however, less than 20 % of these mice were able to resist a subsequent tumor challenge. Similarly, 4T1 tumor-bearing mice whose tumors were eradicated by treatment with the combination therapy were better able to resist subsequent tumor challenge when compared to mice whose tumors were treated with 132 J/cm^2^ (Fig. 4b; *P* ≤ 0.01). Since the immune-enhancing treatment regimen did not eradicate 4T1 tumors in any animals, it was not possible to measure subsequent resistance to 4T1 tumor challenge in animals treated with immune-enhancing PDT regimens.

**Fig. 5:**
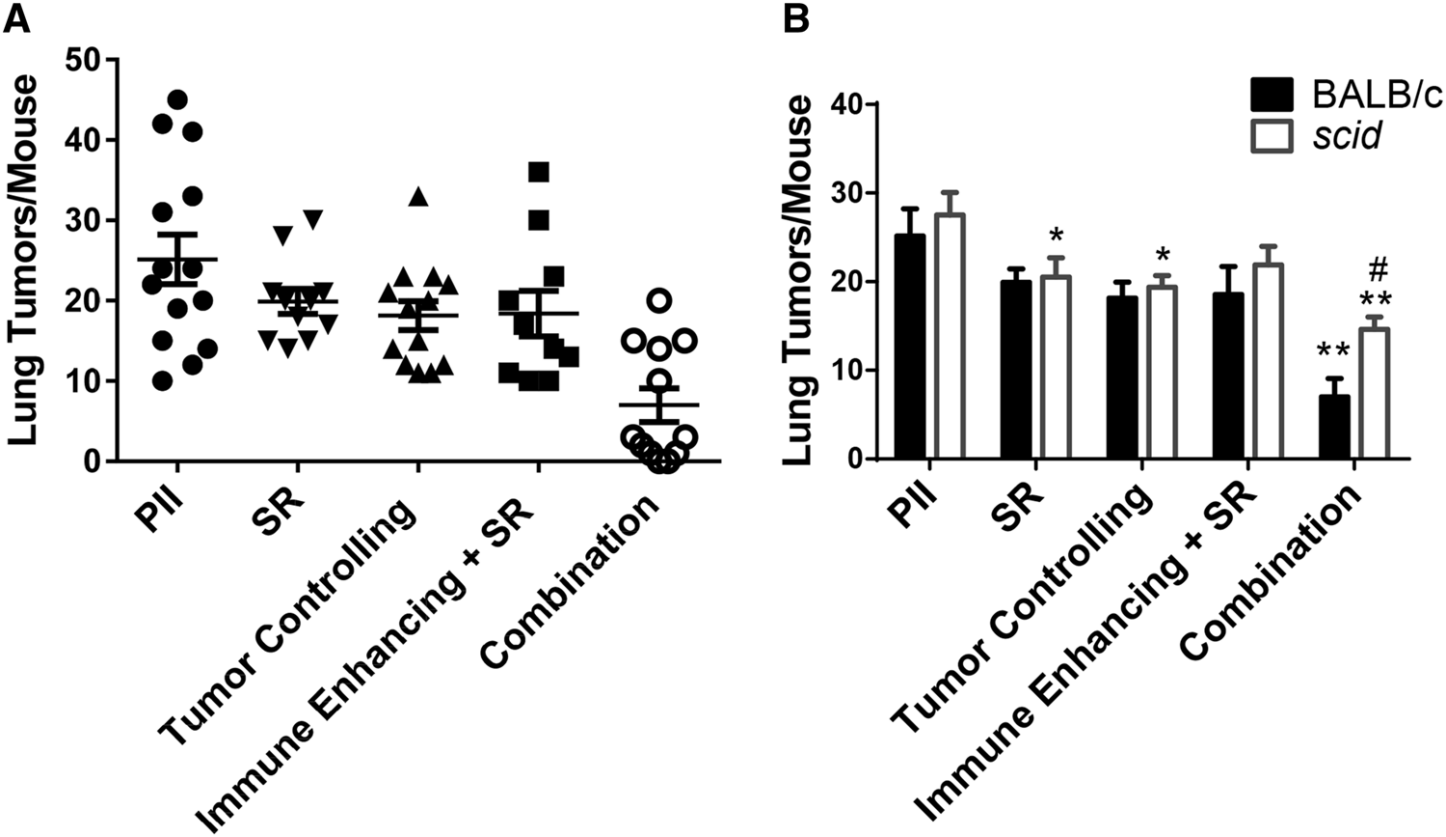
Combination therapy reduces 4T1 lung metastases. 4T1 tumor-bearing BALB/c (**a** **+** **b**) or *scid* (**b**) mice were treated with immune-enhancing PDT as described in Fig. 2. After 9 days, treated mice were re-injected with PII (5 mg/kg); 18–24 h later tumors were either surgically removed (Immune Enhancing + SR) or treated with tumor-controlling PDT (combination). Tumors at an equivalent size to those present in mice treated 10 days prior with the immune-enhancing PDT regimen were treated with PII alone, surgically removed (SR) or treated with the tumor-controlling PDT regimen (tumor controlling). Lungs were collected 14 days later, and the number of metastases/lung was determined. **a** Results represent the number of tumors tumors/mouse. *Each symbol* represents an individual mouse. The mean is represented by a *line,* and the *error bars* represent the SEM. Each group contains a minimum of ten mice. **b** Results represent the average number of lung tumors/mouse. *Error bars* represent SEM; each group contains a minimum of ten mice. *P ≤ 0.05, **P ≤ 0.001 when compared to treatment with PII alone; ^#^ *P* ≤ 0.05 when compared to BALB/c

The increase in effectiveness and anti-tumor immunity observed following treatment of 4T1 tumors with the combination therapy suggests that this treatment regimen may control metastatic tumor growth. The 4T1 tumor rapidly metastasizes to the lung [25]. To assess the effect of PDT regimens on metastasis, 4T1 tumor-bearing mice were treated with immune-enhancing PDT. After 9 days, mice that had been treated with immune-enhancing PDT regimens were injected with PII. The following day, previously treated tumors were either surgically removed (Immune Enhancing + SR) or retreated with tumor-controlling PDT (Combination). Tumors at an equivalent size to those present in mice treated 10 days prior with the immune-enhancing PDT regimen were treated with PII alone, surgically removed (SR) or treated with the tumor-controlling PDT regimen (Tumor Controlling). Lungs were collected from the treated and control mice 14 days later, and the number of metastases/lung was determined (Fig. 5a).

Neither surgical removal of 4T1 tumors, treatment with tumor-controlling PDT, nor treatment with immune-enhancing PDT regimen followed by surgical removal of tumors had a significant effect on the number of tumors present in the lung when compared to treatment with PII alone. In contrast, treatment with the combination therapy significantly reduced the number of tumors present in the lung compared to all other treatments tested (*P* ≤ 0.02).

To determine whether the reduction in lung metastases observed following the combination therapy was a result of enhanced anti-tumor immunity, the combination treatment was carried out in immune-compromised *scid* mice (Fig. 5b). No significant difference was observed between the numbers of lung tumors present in immune-competent BALB/c versus the number present in immune-compromised *scid* mice 14 days after surgical removal of the primary 4T1 tumor or treatment of the primary tumor with either PII alone, tumor-controlling PDT, or immune-enhancing PDT followed by surgical removal of the primary tumor. However, surgical removal and treatment of the primary tumor with the tumor-controlling PDT regimen did significantly reduce the number of lung tumors present in *scid* mice when compared to treatment with PII alone (*P* ≤ 0.05). Thus, it appears as though the reduction in lung tumors observed following surgical removal of the primary tumors or treatment of the primary tumors with tumor-controlling PDT was not a result of enhanced anti-tumor immunity.

The number of lung tumors present in *scid* mice was significantly higher than those present in BALB/c mice following treatment of primary 4T1 tumors with the combination PDT regimen (Fig. 5b; *P* ≤ 0.03); suggesting that anti-tumor immunity plays a role in control of metastatic tumor growth following treatment. However, the combination therapy was still able to control metastatic growth in the absence of a functioning adaptive immune system as the number of lung tumors present in *scid* mice following the combination therapy was significantly lower than the number of lung tumors present following surgical removal of the primary tumor (*P* ≤ 0.04) or treatment or the primary tumor with PII alone (*P* ≤ 0.0002), tumor-controlling PDT (*P* ≤ 0.02), or immune-enhancing PDT followed by surgical removal of the primary tumor (*P* ≤ 0.01).

## Discussion

Recent studies have shown that the host response to PDT is influenced by the treatment parameters [2] and that treatments of different parameters can be used to in combination to achieve desirable outcomes [26]. In the current study, we combine an immune-enhancing PDT regimen with a tumor growth-controlling PDT regimen in order to develop a single modality combination therapy that would both control primary tumor growth and stimulate the host immune system to modulate growth of distant disease. Treatment of tumor-bearing mice with this novel combination therapy resulted in efficacious tumor growth control, increased numbers of activated CD8^+^ T cells and tumor-specific cytotoxicity, and an increased in resistance to subsequent tumor challenge and metastatic tumor growth. Enhancement of anti-tumor immunity by the combination treatment regimen occurred when either HPPH- or PII-PDT was used. Additionally, colon and mammary carcinoma models exhibited regimen-dependent host responses. This suggests that these findings may be applicable across photosensitizers and that it may be possible to identify immune-enhancing PDT regimens in multiple tumor models.

The mechanism of the dose-dependent induction of inflammation and subsequent enhancement of anti-tumor immunity by PDT is unclear. Previous studies by Henderson et al. [2] have suggested that treatment of tumors with high-dose PDT regimens result in vascular ablation that prevents infiltration of the tumor bed by innate immune cell and limits acute inflammation. Initiation of inflammation and subsequent stimulation of anti-tumor immunity have been linked to the recognition of alarmins by innate immune cells [27, 28]. Alarmins include damage-associated pattern proteins (DAMPs) and metabolites released or expressed on the cell surface by damaged and transformed cells, as well as cytokines such as IL-1α. Alarmins are released following PDT and have been linked to generation of inflammation and anti-tumor immunity [29–32]. Recent studies by Tracy et al. [33] have demonstrated that PDT dose influences the release of alarmins. Significantly at high doses, in vitro PDT treatment of tumor cells leads to an inactivation of alarmins and a reduction in the release of inflammatory mediators by stromal cells. In contrast, Garg et al. [34] showed that expression of a cell surface-associated DAMP, calreticulin, increased with PDT dose; however, the ability of ecto-calreticulin induced by high-dose PDT to induce inflammation was not tested.

Once activated in the lymph node, CD8^+^ T cells become either effector or memory cells [35]. Effector cells leave the lymph node and migrate to the tumor site [36]; memory cells migrate to secondary lymphoid organs (central memory T cells) or non-lymphoid tissues (effector memory T cells) [36, 37]. We have previously shown that there is an increase in activated tumor-specific CD8^+^ T cells in the treated tumor bed 7 days after treatment with the immune-enhancing PDT regimen [5]. Activated, proliferating T cells are susceptible to PDT [23]; therefore, it was possible that treatment of tumors with a second PDT regimen 10 days after treatment with the immune-enhancing PDT regimen would result in elimination of tumor-specific T cells, which may prevent resistance to subsequent tumor challenge. However, mice treated with the combination therapy were able to control tumor growth when challenged 60 days following treatment, demonstrating a persistence of anti-tumor immunity and strongly suggesting the presence of long lived memory T cells.

Importantly, the enhancement of immune reactivity to Colo26 tumors by either the immune-enhancing or combination PDT regimens was not limited to recognition of an over-expressed surrogate antigen, confirming our previous preclinical and clinical studies demonstrating that PDT enhances immune recognition of endogenous tumor antigens [5, 38]. Similarly, Mroz et al. [39] showed that vascular PDT regimens enhanced immune reactivity against the endogenous cancer/testis antigen PA1; although this treatment regimen was unable to augment anti-tumor immunity against endogenous tumor antigens in Colo26 tumors.

Treatment of either the colon or mammary tumor model with the combination therapy resulted in effective control of primary tumor growth; in the case of the Colo26-HA tumor model, there was no significant difference in primary tumor growth following treatment with the tumor growth-controlling PDT regimen and the combination therapy regardless of the photosensitizer used. In contrast, the combination therapy controlled primary 4T1 tumor growth significantly better than single treatment with the tumor-controlling PDT regimen. The reason for this difference is unclear. One possibility is that although tumor size was controlled such that the tumors treated with the single dose of tumor-controlling PDT regimen were of similar size to those treated 10 days following the immune-controlling PDT regimen in the combination therapy, the immune-controlling PDT regimen sensitized the orthotopic tumors to PDT in some manner. The immune-controlling PDT regimen induces a strong local inflammatory response, which could be responsible for this alteration. Studies are currently underway to address this possibility.

Orthotopic 4T1 tumors rapidly metastasize to the lung and liver within 21 days of inoculation [25]. We and others have previously shown that PDT-enhanced anti-tumor immunity can control distant disease [8, 40–42]. The combination therapy was also able to control 4T1 tumor metastases; although the control was not entirely dependent upon the immune system as control was significantly but not completely ablated when the combination therapy was performed in *scid* mice. Metastatic tumor growth was also partially controlled following surgical removal of the primary tumor and treatment with tumor-controlling PDT regimens; this control was independent of anti-tumor immunity. The number of lung tumors present increases with the size of the primary 4T1 and the amount of time the tumor is present in the animal [25]. Therefore, it is possible that the adaptive immune system-independent reduction in metastatic tumor growth is due to the absence of the primary tumor following surgical removal or treatment-induced reduction in primary tumor growth following treatment with the tumor growth-controlling PDT regimen.

Our studies support the use of an immune-enhancing PDT regimen as an adjuvant treatment that, when used in conjunction with therapies that inhibit or do not enhance immunity, has the potential to augment anti-tumor immunity. Recent studies have shown that PDT is an effective adjuvant therapy to surgery that increases the probability of long-term local disease control [1]. Friedberg and colleagues have demonstrated prolonged survival in malignant pleural mesothelioma patients treated with radical pleurectomy and intraoperative PDT [43] that the authors attribute in part to the immune effects of PDT.

In summary, we have identified an anti-tumor combination PDT treatment regimen that controls primary and metastatic tumor growth and enhances anti-tumor immunity. The combination therapy consists of treatment with an immune-enhancing PDT regimen followed 10 days later by treatment with a tumor growth-controlling treatment regimen. Treatment of both colon and mammary carcinomas showed similar host responses, and the host responses were independent of the photosensitizer used. These findings provide proof of principle for the use of PDT as an adjuvant therapy for enhancement of anti-tumor immunity that may be capable of controlling distant disease.

## Abbreviations

CTL: Cytotoxic T lymphocyte
CSFE: Carboxyfluorescein triacetate, succinimidyl ester
DAMP: Damage-associated pattern protein
HA: Hemagglutinin
HPPH: 2-[1-Hexyloxyethyl]-2-devinyl pyropheophorbide-a
IACUC: Institute Animal Care and Use Committee
MHC: Major histocompatibility complex
PDT: Photodynamic therapy
PII: Sodium Porfimer
TDLN: Tumor-draining lymph node
